# Integrating Social Justice into Higher Education Conservation Science

**DOI:** 10.1093/biosci/biac008

**Published:** 2022-03-30

**Authors:** Robert A Montgomery, Abigail M Pointer, Sophia Jingo, Herbert Kasozi, Mordecai Ogada, Tutilo Mudumba

**Affiliations:** Department of Zoology, University of Oxford, Oxford, England, United Kingdom, and with The Recanati-Kaplan Centre, Tubney, England, United Kingdom; Michigan State University, East Lansing, Michigan, United States; Michigan State University, East Lansing, Michigan, United States; Michigan State University, East Lansing, Michigan, United States; Conservation Solutions Afrika, Nanyuki, Kenya; Michigan State University, East Lansing, Michigan, United States; Department of Zoology, Entomology, and Fisheries Sciences, Makerere University, Kampala, Uganda

**Keywords:** biodiversity, conservation, education, interdisciplinary science, social justice

## Abstract

Because biodiversity loss has largely been attributed to human actions, people, particularly those in the Global South, are regularly depicted as threats to conservation. This context has facilitated rapid growth in green militarization, with fierce crackdowns against real or perceived environmental offenders. We designed an undergraduate course to assess student perspectives on biodiversity conservation and social justice and positioned those students to contribute to a human heritage-centered conservation (HHCC) initiative situated in Uganda. We evaluated changes in perspectives using pre- and postcourse surveys and reflection instruments. Although the students started the course prioritizing biodiversity conservation, even when it was costly to human well-being, by the end of the course, they were recognizing and remarking on the central importance of social justice within conservation. We present a framework for further integration of HHCC approaches into higher education courses so as to conserve the integrity of coupled human and natural systems globally.

Although its roots lie in long-established fields, including ecology, population biology, and genetics, conservation biology was not established as an independent discipline until the 1970s (Soulé and Wilcox 1980, Van Dyke and Lamb 2020). The discipline was created to conduct empirical research into the mechanisms associated with biodiversity loss and to use that information to develop best practices to evidentially conserve biodiversity (Soulé 1985, 1986, Trombulak et al. 2004). Since its inception, dramatic environmental changes and a number of highly influential wicked problems have challenged the ability of conservation scientists and practitioners to achieve the mandate of the second founding goal (Barnosky et al. 2012, Game et al. 2014, Ceballos et al. 2015, Colchero et al. 2019). Emblematic of this point, more than 60% of global terrestrial biodiversity has been lost (see Leclère et al. 2020) since the first conference on conservation biology was hosted in 1978. Furthermore, the pace of biodiversity loss is estimated to be 100 to 1000 times higher than background extinction rates (assumed to be 0.1 to 1 species extinction per million species per year; Ceballos et al. 2015). This has led many to suggest that the sixth mass extinction event—and the first principally accelerated by human action—has already begun (Wake and Vredenburg 2008, Barnosky et al. 2011, Pimm et al. 2014).

The factors contributing to biodiversity loss include habitat fragmentation, disease, climate change, conflict, invasive speciation, overhunting, and guild disruption (Forester and Machlist 1996, Newbold et al. 2015, 2016, Macdonald 2016, Maxwell et al. 2016, Horváth et al. 2019). As many of these factors are initiated or mediated by human actions, people are regularly depicted as threats to biodiversity conservation (Dirzo et al. 2014, Ceballos et al. 2015). The presentation of conservation as a global responsibility (e.g., among the sustainable development goals of the United Nations; UN 2015) and a “crisis discipline” (Soulé 1985) have created strong subtexts about the intensive measures necessary to protect biodiversity (Schultz 2011, Lennox et al. 2020). Such “conserve at all costs” mentalities, however, have not only been found to decrease the credibility of conservation science (Komonen et al. 2019) but also to engender circumstances in which biodiversity conservation has been prioritized over basic human rights (Chan 2008, Mbaria and Ogada 2016).

Consequently, conservation science has come to be defined by a number of value-based dilemmas involving the ideals of biodiversity protection and those of social justice (Stern et al. 1999, Fornara et al. 2020). These dilemmas are perhaps most obvious and apparent in the Global South (Brooks et al. 2006, Habel et al. 2019), where conservation tends to be harshly dichotomized between the preservation of biodiversity and human well-being (Happold 1995, Redford et al. 1998, Schwartzman et al. 2000). Strategies designed to protect biodiversity, for example, tend to focus on curbing human consumption, much of which occurs illegally in the rural communities adjacent to biodiverse-rich areas (Berkes 2004, 2007). This context has facilitated widespread growth in the militarization of conservation (Massé and Lunstrum 2016, Duffy et al. 2019), which has been associated with fierce crackdowns against people perceived to be guilty of environmental crimes (Lunstrum 2014, Annecke and Masubelele 2016, Warren and Baker 2019, Warren et al. 2019). Although it is undoubtable that human actions regularly contribute to the decline of biodiversity, people can experience profoundly negative impacts via the implementation of conservation practice (Chapin et al. 2000, Cardindale et al. 2012, Isbell et al. 2017).

Conservation and sustainability are highly pertinent issues among the students presently enrolling in higher education institutions around the world (O'Brien et al. 2018, Rosin and Zedler 2020, Barbiroglio 2019). However, the extent to which students value social justice within conservation remains unclear. We designed a semester-long (i.e., 16-week) course to expose a group of 21 undergraduate students enrolled in a public university in the United States to emergent designs in biodiversity conservation, focusing on the human heritage-centered conservation (HHCC) framework (see Montgomery et al. 2020). The intention of this course was to teach the students how to unpack global problems in conservation and to highlight practices that could demonstrably protect biodiversity while uplifting the livelihoods of local people. We implemented a pre- and postcourse survey to evaluate student perspectives of the dualities of biodiversity conservation and social justice. We hypothesized that, at the outset of the course, the students would value biodiversity, even when the implementation of conservation practice came as a detriment to local people. We also hypothesized that more balanced viewpoints of the role of social justice in conservation would be evident in the postcourse survey. We detail the course design so as to facilitate replication across higher education institutions and discuss the importance of HHCC approaches in conservation science. We also describe the ways in which undergraduate students can be mobilized to make global impacts from campus via tangible distance-learning experiential designs.

## Human heritage-centered conservation framework

The HHCC framework departs from community-based conservation, which—in part because of its overly general terminology—has experienced dramatic variation in interpretation and application (see Montgomery et al. 2020). In place, the HHCC framework positions the cultural heritage of local people at the heart of conservation practice. The framework demonstrates that people and nature are not separate entities but, rather, integral components of coupled human and natural systems. The HHCC framework is defined by a set of 10 tenets (see table 1; Montgomery et al. 2020). These tenets describe the actions that practitioners must embrace to promote alignment between the implementation of conservation practice and the heritage of local human communities. The framework embraces indigenous languages; incorporates local people, agencies, and organizations into symbiotic collaborations; promotes interdisciplinarity; and provides a series of robust recommendations to promote the professional development of people residing in communities adjacent to biodiverse-rich areas (table 1). All of these efforts serve to sustain conservation practices over time via ethical and mutually beneficial partnering between conservation practitioners and members of local human communities.

**Table 1.** The ten tenets defining the human heritage-centered conservation framework as defined by Montgomery and colleagues (2020).

Tenet	Tenets of human heritage-centered conservation
1	Engage in conservation practices using local languages
2	Incorporate traditional ecological knowledge into conservation practices
3	Foster interdisciplinary research teams to develop novel conservation solutions
4	Collaborate with local environmental authorities in research-informed conservation
5	Thoughtfully propose and apply solutions that are consistent with human heritage
6	Present clear professional development opportunities for employees from local communities
7	Provide educational and technical training to people from local communities
8	Facilitate terminal degree training pathways for students from local communities
9	Promote alternative revenue-generating programs centered in local communities
10	Develop peer-reviewed evidence of the efficacy of the conservation solutions

## Higher education course design

We prepared a course built on the HHCC framework to enable undergraduate students at Michigan State University, a large (more than 50,000 undergraduate students) higher education institution in the Midwestern region of the United States, to explore values of social justice and biodiversity conservation. We designed the course as an upper-division offering while making it an elective without prerequisites so as to broaden enrolled student diversity both in terms of disciplinary major and time to degree completion. Meeting twice a week, over 2 hour and 50 minute periods, we offered the course across the 16-week spring semester of 2019, with the enrolled students receiving four credits. In framing the course content, we focused on case studies involving with the interactions of local people and biodiversity conservation in East Africa. We also enabled the students to engage with the social justice dimensions of conservation via participation in an HHCC project based in Uganda called the Snares to Wares Initiative.

We divided the course into three units, including burn-in (2 weeks), orientation to the Snares to Wares Initiative (2 weeks), and key-performance periods (four periods each covering 3 weeks). During the burn-in unit, we taught the students how to unpack global conservation problems. Within that process, we assigned the book The Big Conservation Lie as a required course text (Mbaria and Ogada 2016). In this book, Mbaria and Ogada (2016) explored several conservation paradigms, portrayed via personal experience and investigative research, throughout the East African region, highlighting the important social justice dimensions inherent to conservation. With the students embracing the complexities of conservation, we next oriented them to an HHCC project called the Snares to Wares Initiative (described below). During this 2-week unit, we used short (i.e., less than 20 minutes) lectures to provide context regarding the mission and values of the initiative, implemented break-out sessions to promote student dialogues, and entertained all-class discussions to synthesize concepts. Finally, we had four key-performance periods, each lasting 3 weeks, in which the students worked cohesively in teams of between four and five students to produce deliverables with tangible outputs for the Snares to Wares Initiative.

## The snares to wares initiative

**Figure 1.**
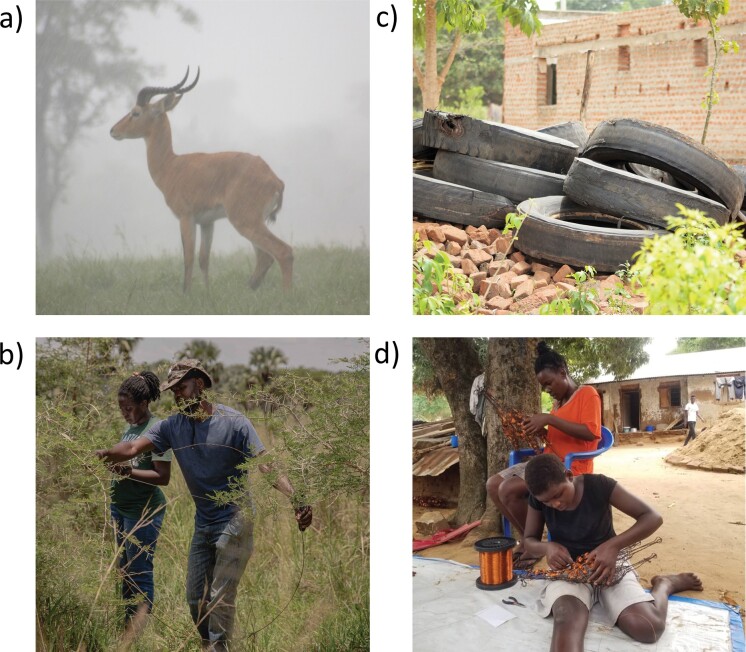
The Snares to Wares Initiative is an intervention designed to conserve biodiversity by uplifting local human communities in conservation. In Murchison Falls National Park, Uganda subsistence poaching not only represents a conservation problem, but also an entrenched social justice issue. The Uganda kob (Kobus kob) is one of the primary targets of subsistence poachers (a). Peter Luhonda and Sophia Jingo removing wire snares from the national park (b). The wires used by subsistence poachers in this region often derive from radial vehicle tires (c). The local artisans of the Snares to Wares Initiative produce bespoke pieces representing animals that are subjected to poaching pressure (d). Photographs (a), (d) Tutilo Mudumba; (b), (c) Esther Ruth Mbabazi.

We used distance-learning approaches to enable the students to participate in the Snares to Wares Initiative, which is situated in the communities adjacent to Murchison Falls National Park, Uganda. The initiative is an intervention enabling local people to tangibly benefit from biodiversity conservation. Abject poverty, the lack of alternative livelihood options, and conflict with wildlife have led many in these communities to illegally harvest wildlife for subsistence. Although antelopes are often the intended targets (figure 1a), the wire snares (originating from disused radial vehicle tires) that are used to poach are indiscriminate and capture a number of species of conservation concern (Mudumba et al. 2021). Furthermore, poaching in this landscape is a highly risky activity in which the perpetrators are subject to substantial penalties, including long-term prison sentences and even shoot-on-site provisions. The Snares to Wares Initiative removes these snares from the national park (figure 1b), collects abandoned vehicle tires to prevent their wires from entering the national park (figure 1c), provides artisanal training to enable local people (many of whom are reformed poachers) to repurpose the wires into works of art (figure 1d), and creates markets to sell that art both locally and internationally, generating revenue and employment to uplift these local human communities in conservation. We enabled the students to work in partnership with local people in Uganda in this HHCC intervention via the development of working teams.

## Team membership

We determined team membership using two criteria: responses to a personality test and student rankings of their team preference. The Smalley Trent Personality Test (see appendix A in the supplemental material) consists of 10 questions to determine whether the students were lions (dominance), otters (influence), beavers (compliance), golden retrievers (steadiness), or some combination of the above. While considering preference for the teams, we administered the personality test to ensure that each team had diversity in student willingness to lead, to follow directions, and to resolve problems.

We created four teams contributing to marketing and engagement (i.e., content development, donor outreach), graphical data representation (i.e., data analysis, presentation, and writing), business and value chain (i.e., e-commerce, shipping, and distribution), and broader impacts (i.e., event management, community engagement, and sustainability of the initiative; see appendix A in the supplemental material). Although these team identities provided broad framing on the types of outcomes needed, the actual activities pursued were identified and selected by the students themselves. These teams worked together across the remainder of the semester, although collaborative work among multiple teams was encouraged and produced deliverables at the conclusion of each period. We assessed these deliverables according to their demonstrated quality, creativity, ingenuity of design, and potential for positive impact on the Snares to Wares Initiative.

## Evaluation of student perspectives

To evaluate the students’ perspectives of biodiversity conservation and social justice, we implemented a pre- and postcourse survey (see appendix B in the supplemental material) administered on the first and final days of the course (i.e., separated by 16 weeks). We made clear that the survey was not mandatory and that the students could opt out if they were so inclined. When developing the instrument, we evaluated surveys in the peer-reviewed literature and adapted portions of the published questionnaire developed by Jacobsen and Linnell (2016). We were drawn to this instrument, in particular, because it evaluated the roles and responsibilities of various people (i.e., local people, conservationists, and management authorities) within the context of human–wildlife conflict. Conflict occurs when human–wildlife interactions yield negative outcomes for one or both parties (Montgomery et al. 2022). People can experience negative outcomes from wildlife interaction including risks to human security and private property (i.e., crop raiding, food insecurity, livestock depredation, and damage to infrastructure; Redpath et al. 2013, Montgomery et al. 2022). In response to these risks, people may retaliate against wildlife in the form of killing or poaching, both of which are illegal (Moreto 2019). In these ways, conflict with wildlife is not only a key contributor to biodiversity loss (Milner-Gulland and Bennett 2003, Ripple et al. 2019) but also a central social justice issue in conservation (Steinhart 2006, Duffy 2014, 2019). Therefore, structuring the course content around human–wildlife conflict and subsistence poaching enabled us to evaluate the students’ perspectives of biodiversity conservation versus social justice and how those valuations changed over the semester.

We divided the survey questions into four categorical sections assessing the students’ values of wildlife, views about who has responsibilities for wildlife, views about appropriate responses to negative outcomes from wildlife, and views about appropriate solutions for such conflict. We asked the students to rate their level of agreement of each statement on a five-point Likert scale, ranging from strongly agree (1) to strongly disagree (5). Finally, we concluded the survey with demographic data on student gender, grade level, age, and major. To assess variation in the students’ responses between the pre- and postcourse surveys, we used a paired t-test and interpreted significant changes on the basis of the α ≤ .05 level. All protocols for evaluation of the students’ perspectives were approved by Michigan State University's Institutional Review Board (IRB no. X17-831e).

## Controlling for potential euphoria bias

We provided the students with an immersive and engaging experience to make global impacts without ever leaving campus. Such experiential learning opportunities are prone to euphoria bias, where the students are so impressed by the experience that their assessments are positively biased (Marsh et al. 1986). To control for potential euphoria bias (see Austin et al. 2009), we asked the students to develop short (less than 700 words) reflection documents at the start of the course (following the burn-in period) and at the end of the course to convey the things that they were thinking about, what they found enjoyable about the course, and what they found challenging. We then conducted a thematic analysis of the frames featured in these reflection documents following the methods outlined by Wolter and colleagues (2011). Specifically, four of the present authors independently read the student reflection documents, which we anonymized to protect the students’ identities. Each of the four coauthors generated a list of the emergent themes that were evident in the start and end of course reflection documents. The four coauthors then compared their emergent theme lists. Via this process, we developed a list of predominant themes from the start of course reflections and the end of course reflections. All four coauthors then reread the reflection documents and quantified the frequency with which themes were referenced by the students.

## Student Perspectives

The course featured 17 female students and 4 male students pursuing eight different academic majors (table 2). The students also varied with respect to time to degree with seven sophomores, four juniors, and nine seniors (table 2). Among the 21 students, a total of 20 completed both pre- and postcourse surveys for a matched-pairs response rate of 95%. The same response rate (95%; n = 20 of 21) was evident among the start of course reflections, with a 90% (n = 19 of 21) response rate for the end of course reflection.

**Table 2.** Demographic characteristics of the 20 students enrolled in the human heritage-centered conservation course that completed both the pre- and postcourse surveys.

Demographic category	Demographics	Age (in years)	Proportion
Age	Mean age	20.5	–
	Age range	19–23	–
Gender	Male	–	.20
	Female	–	.80
Grade level	Freshman	–	0
	Sophomore	–	.40
	Junior	–	.20
	Senior	–	.40
Discipline	Fisheries and Wildlife	–	.30
	Packaging	–	.20
	Zoology	–	.15
	Animal Science	–	.10
	Business	–	.05
	Environmental Science and Sustainability	–	.05
	Sustainable Parks, Recreation, and Tourism	–	.05
	Professional Writing	–	.05
	Political Science	–	.05

**Figure 2.**
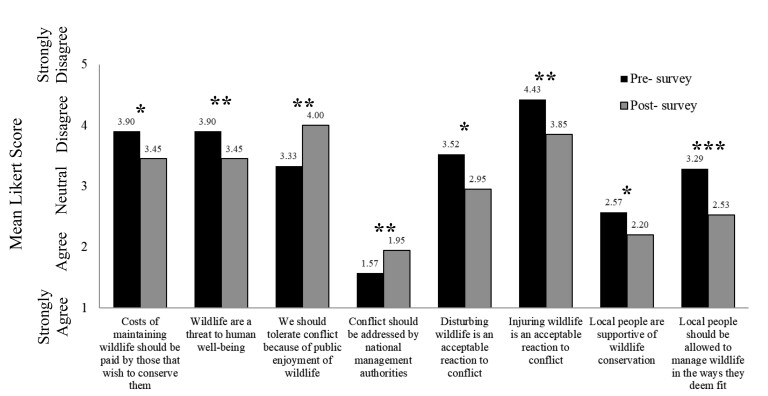
Statistically significant (at α = .05) differences, as measured by paired t-tests, in student responses between the pre- and postcourse surveys of the human heritage-centered conservation course.

In comparing the pre- and postcourse surveys via a paired t-test, we detected statistically significant (at α ≤ .05) changes in the student responses across almost a third (27%, n = 8 of 30) of the questions (see figure 2). Please see appendix C in the supplemental material for the nonsignificant student responses. Among the significant changes, the students shifted from disagree, in the precourse survey, to neutral, in the postcourse survey, when asked if the costs of maintaining wildlife should be paid by those that wish to conserve them (t(19) = 2.39, p = .03; figure 2). A similar shift from disagree to neutral was evident when the students were asked whether wildlife are a threat to human well-being (t(19) = 3.60, p = .002; figure 2). The students shifted from neutral, in the precourse survey, to disagree, in the postcourse survey, when asked whether people should tolerate conflict with wildlife because of public enjoyment of wildlife (t(19) = –3.04, p = .007; figure 2). The students shifted from strong agreement, in the precourse survey, to agreement, in the postcourse survey, when asked whether conflict with wildlife should be addressed by national management authorities (t(19) = –3.20, p = .005; figure 2). When asked whether disturbing wildlife was an acceptable reaction to conflict, the students shifted from disagree, in the precourse survey, to neutral in the postcourse survey (t(19) = 2.60, p = .02; figure 2). A similar shift from disagree to neutral was evident when the students were asked whether injuring wildlife was an acceptable reaction to conflict (t(19) = 2.98, p = .008; figure 2). The students shifted from neutral, in the precourse survey, to agreement, in the postcourse survey, when asked whether local people are supportive of wildlife conservation (t(19) = 2.65, p = .016; figure 2). Finally, when asked whether local people should be allowed to manage wildlife in the ways that they deem fit, the students shifted from neutral, in the precourse survey, to agreement, in the postcourse survey (t(19) = 4.03, p < .001; figure 2).

There were 123 coded responses in the start of course reflections and from those, we identified five predominant themes, including enlightenment, discomfort, pathway, motivation, and inclusion (table 3). Enlightenment (33%; table 3) was the most common theme, where the students articulated that their minds were opened to new information via participation in the course. One student commented that, “This course has opened me up to a new way of thinking, pushing me to analyze all that I have previously learned and believed” (table 4). Discomfort (30%; Table 3) was a close second, with one student commenting that, “Finding out that I didn't know the real truth behind conservation efforts was a blow to my morale and confidence of the field” (table 4). The students also acknowledged that the course served as a pathway (15%; table 3) to tangibly contribute to conservation and sustainability. One student wrote, “After approaching every class I've ever taken as a student, the chance to contribute to a project creating a real positive impact within a community is exciting” (table 4). Just under a quarter (13%; table 3) of the coded student responses described the students’ motivation and enthusiasm in enrolling in this course. One student wrote, “I became very interested in being involved in some capacity with conservation as it relates to the Global South” (table 4). Finally, 9% of the coded responses referred to inclusion (table 3), not only relating to student demographic characteristics but also to disciplinary domains. One student wrote, “It is so interesting to be surrounded by students of all ages and disciplines, because even though I attend a large university, I hardly interact with students outside of my department or college. I feel as if many other courses could benefit from a more interdisciplinary and open environment” (table 4).

**Table 3.** Quantification of the number of times and proportion that each theme was referenced in the start of course and end of course reflections.

Survey	Main theme	Count of themes (n)	Proportion
Start of course	Enlightenment	41	.33
	Discomfort	37	.30
	Pathway	18	.15
	Motivation	16	.13
	Inclusion	11	.09
End of course	Development	53	.33
	Enjoyable	34	.21
	Commitment	27	.17
	Frustration	26	.16
	Contributions	19	.12

**Table 4.** The main themes deriving from student reflections in the start of course and end of course surveys.

Survey	Main theme	Description	Representative student quote
Start of course	Motivation	An interest to contribute to conservation and sustainability	“I became very interested in being involved in some capacity with conservation as it relates to the Global South.”
	Enlightenment	Exposed to new content for the first time that altered ways of thinking	“This course has opened me up to a new way of thinking, pushing me to analyze all that I have previously learned and believed.”
	Discomfort	Exposure to new content and ways of thinking was emotionally charged	“Finding out that I didn't know the real truth behind conservation efforts was a blow to my morale and confidence of the field.”
	Pathway	The course acting as a tangible pathway facilitating student engagement in conservation and sustainability	“After approaching every class I've ever taken as a student, the chance to contribute to a project creating a real positive impact within a community is exciting.”
	Inclusion	The course acting as a means to navigate issues of inclusivity among racial, disciplinary, and societal divides	“It is so interesting to be surrounded by students of all ages and disciplines, because even though I attend a large university, I hardly interact with students outside of my department or college. I feel as if many other courses could benefit from a more interdisciplinary and open environment.”
End of course	Frustration	Frustrations related to the difficult of the course, the challenges of working in teams, issues associated with distance learning, and sensations of wishing to accomplish more across the semester	“It can be difficult to get started and really understand what this class is about.”
	Enjoyable	Impressions of the course being novel, original, fun, and disbelief at how quickly each class period lasted	“As the last day of class has approached, I am in disbelief of the time that has flown by this semester.”
	Development	Impact of the course on student learning, skill development and transfer to future career outcomes	“This class has been a really good way to get involved in things I haven't done before and given me skillsets that I can apply to research going forward.”
	Contributions	The course made tangible contributions people and wildlife living in real-world systems	“Realizing that what I have done here has an impact on people over in Pakwach is surreal, especially because I feel like undergraduate students are not expected to do things that are meaningful.”
	Commitment	Student commitment to continue contributing to people and wildlife in these real-world systems and representing the learning outcomes among family, friends, and broader community	“I want to carry on telling the story of Snares to Wares and how wire snares affect the lives of the wildlife and the people of East Africa.”

*Note:* Descriptions of those themes along with student quotes that exemplify each point are provided.

In the end of course reflections, there were 159 coded responses and five predominant themes, including development, enjoyable, commitment, frustration, and contributions. The most common code was development, which occurred in 33% of the responses (table 3). Within this context, the students recognized that the course learning materials provided skills (communication, empathy, and team building) that transferred to professional workplaces. Approximately 21% of the coded responses described how enjoyable the students found the course (table 3). One student wrote, “As the last day of class has approached, I am in disbelief of the time that has flown by this semester” (table 4). Enjoyment of the class seemed to be connected with student commitment, referenced among 17% of the coded responses (table 3). Within this context, one student wrote, “I want to carry on telling the story of Snares to Wares and how wire snares affect the lives of the wildlife and the people of East Africa” (table 4). The course did create a series of frustrations, expressed in 16% of the coded responses (table 3), associated with difficulties in working in teams, experiencing failure in realizing various goals within their key-performance periods, and the students’ perceptions of wishing they could accomplish more over the semester. Finally, in 12% of the coded responses, the students spoke about the contributions that they made in the course (table 3). As one student wrote, “Realizing that what I have done here has an impact on people over in Pakwach [Uganda] is surreal, especially because I feel like undergraduate students are not expected to do things that are meaningful” (table 4).

## Implications

Although humans have been integral components of ecological systems for tens of thousands of years (Milner-Gulland and Bennett 2003, Bird and Nimmo 2018, Ellis et al. 2021), historic and current research has shown that people often envision themselves to be decoupled from the natural world (Tansley 1935, Alberti et al. 2003, Moll et al. 2021). Such disassociations, however, engender false notions about humans and nature being mutually exclusive spheres (Redford and Sanderson 2000, Waldron et al. 2017). Consequently, in an effort to protect biodiversity people—and particularly so in the Global South—are often coarsely depicted as threats to conservation as evidenced by the rapid growth of both the fortress and militarization of conservation (Duffy et al. 2019, Montgomery et al. 2020). These conservation paradigms, however, have exposed considerable violations to human rights underscoring the fundamental need to integrate social justice into conservation practice (Chan 2008, Mbaria and Ogada 2016). Although progress has been made to consider the principles of social justice in conservation research (Martin et al. 2016, Bennet et al. 2017), we attest that similar advances must take place in higher education so that conservation teaching also embraces these fundamentally important dualities associated with the ideals of human well-being and biodiversity conservation.

Via the implementation of our HHCC course, we observed significant changes in student perspectives across close to a third of the survey questions that we assessed. In the precourse survey, the students disagreed with the notion that the costs of maintaining wildlife should be paid by those that wished to conserve them. This perspective shifted to neutral in the postcourse survey indicative of emergent appreciations that conservation costs should not be the burden of local people alone (figure 2). Trade-offs are inherent to the implementation of conservation practice, and there are a number of actors who can experience costs within this context, including governments, nongovernmental organizations, conservationists, global citizens, and local people (Hirsch et al. 2011). Local people often face both visible (e.g., crop or infrastructure damage and livestock depredation) and hidden (e.g., lack of opportunities or displacement) costs from living in biodiverse-rich areas (Barua et al. 2013). In addition, negative interactions with wildlife or disenfranchisement via the implementation of various conservation practices can also exert nonmaterial costs, in the form of psychosocial effects including trauma and anxiety, for local people (Thondhlana et al. 2020). By the end of the course, the students were appreciating the impact of such costs on the willingness of local people to engage in conservation interventions. We also found that at the start of the course, the students did not perceive wildlife to pose a threat to local people. This too shifted to neutral in the end of course survey (figure 2), indicative of the students’ emerging appreciation of the variety of negative consequences that local people can experience from wildlife (Redpath et al. 2013, Montgomery et al. 2022).

In the precourse survey, the students also had a neutral response when asked whether people should tolerate conflict with wildlife because the public enjoy wildlife. By the end of the course, the students shifted their position to disagree (figure 2), demonstrating empathy for local people subject to various conflicts with wildlife and conservation practice. By the end of the course, the students seemed to recognize that there were instances in which local people should be able to defend their security and private property against conflict-causing wildlife. This was evident in the changes from disagreement to neutrality in reference to questions assessing whether disturbing or injuring wildlife was an acceptable reaction to conflict (figure 2). We interpret this shift to illustrate an emergent appreciation among the students that local people should not be uniformly prevented from defense of their security or private property. People have protected their crops and livestock from wildlife for thousands of years (Treves et al. 2006). Although such provisions are a component of certain pieces of legislation in the Global North, they are not often similarly accessible to local people in the Global South, where disturbing or injuring wildlife is often illegal (Dickman and Hazzah 2016). The students also shifted from strong agreement, in the precourse survey, to agreement, in the postcourse survey, when asked whether conflict with wildlife should be addressed by national management authorities (figure 2). Similarly, the students in the precourse survey were neutral with respect to local people are supportive of wildlife conservation and whether those local people should be empowered to manage wildlife in the ways that they deem fit. However, by the end of the course, the students had changed their perspectives and agreed with both of these points (figure 2). We interpret these results to demonstrate student perspectives that local people should be empowered to be active participants in the management process. Interdisciplinary collaborations involving management authorities, conservationists, and local people are one of the tenets of the HHCC framework and are necessary for emergent approaches seeking to position local people at the heart of conservation practice (Montgomery et al. 2022).

The HHCC course that we developed was clearly a challenging one for the enrolled students. When controlling for euphoria bias, we found that the reflection documents revealed the ways in which the students grappled with a range of emotions, from enlightenment associated with the course materials to discomfort when exposed to the hidden costs of conservation practice for many people in the Global South (table 4; Barua et al. 2013, Thondhlana et al. 2020). Therefore, interpretation of the start of course and end of course reflection documents showed that the students both enjoyed and were challenged by this course. For instance, the start of course reflections were characterized by student discomfort relating to the emotional elements of the course materials. One student wrote, “Finding out that I didn't know the real truth behind conservation efforts was a blow to my morale and confidence of the field” (table 4). This information was not only captured via class lectures and discussion but also by first-person accounts when the students interacted with members of the Snares to Wares Initiative during the key-performance periods. It can be emotionally wrenching to hear the first-person accounts of trauma experienced from the implementation of conservation practice (Thondhlana et al. 2020). The students also expressed frustration in the end of course reflection documents. This frustration related to the challenges of working in teams, a common complaint among undergraduates in higher education (Volet and Mansfield 2006), and an inability to accomplish goals within the key-performance periods. This last point was largely attributable to the speed with which the course seemed to pass and was well expressed by one student, who wrote, “As the last day of class has approached, I am in disbelief of the time that has flown by this semester” (table 4). The students that are bored with higher education commonly describe how slow class time seems to pass (Sharp et al. 2017). Therefore, we interpret the students’ perceptions of the rapid speed of the course to be a clear indication that we provided an engaging and applied offering that was well aligned with the students’ motivations for learning.

The students also found the course to be inclusive, to provide them with transferable skills on their pathway to employment, and to enable them to make tangible contributions in real-world communities. These are all essential characteristics of university education. Extensive research has demonstrated the importance of developing inclusive learning environments in higher education, where all student perspectives are welcomed and valued (Moriña 2017, MuGale et al. 2017). Higher education curriculums that are disconnected from the skills and expectations of the professional workplace are among the reasons students transfer out of natural resources departments, such as conservation science (Wolter et al. 2011). Universities are increasingly becoming locations in which productive partnerships with local communities, agencies, and stakeholders can be established to meet positive conservation outcomes across an academic semester (Gladstone et al. 2006, Dunbar et al. 2013). Experiential learning opportunities like these are an excellent way to keep students engaged in the curriculum (Wolter et al. 2011), and applied learning spaces, such as the one that we provided, are associated with higher retention of course information (Montgomery and Millenbah 2011). Correspondingly, we encourage the orientation of conservation education toward the principles of inclusivity, application to real-world problems, and transferrable skills. These address fundamental gaps in the appropriate training of undergraduates to become career conservation biologists (sensu Noss 1997).

We developed an HHCC course that enabled undergraduate students at a large university to make a global impact without ever leaving campus. This outcome was a fundamental component of our vision for inclusivity. Boutique experiential learning opportunities, such as study abroad, are often self-selected by students from financially advantaged backgrounds (Salisbury et al. 2011). Therefore, there is a need to engineer course offerings that replicate the immersive elements of study abroad from campus to enable participation among a broader student representation. Our vision for inclusivity was also manifest via the creation of interdisciplinary student teams with enrolled students from eight different academic majors (table 2). These teams represented our vision for integrating interdisciplinarity into conservation science education (Montgomery et al. 2018, 2020). Using distance-learning approaches, we enabled these students to tangibly engage in—and help grow—an HHCC project situated in human communities adjacent to Murchison Falls National Park, Uganda. The integration of these students into this HHCC initiative indicated that the students’ work not only supported their own professional development but was simultaneously consequential on the communities engaged in this partnership. We see tremendous potential for these principles of student inclusion and instruction to be scaled throughout higher education courses to diversify conservation curriculums. Presently, conservation curriculums tend to be too biology-centric (Gardner 2021). Failure to acknowledge the fundamental social justice dimensions inherent to conservation science provide students with only a fractional understanding of the complexities inherent to conservation. Growth in interdisciplinarity has come to define emergent conservation research frameworks (Martin et al. 2016, Bennet et al. 2017). We now advocate for similar progress to be made in the instruction of conservation science in higher education via incorporation of the distance-learning and HHCC components that we described in the article.

## Supplementary Material

biac008_Supplemental_Files

## Author Biographical

Robert A. Montgomery (robert.montgomery@zoo.ox.ac.uk) is affiliated with the Wildlife Conservation Research Unit in the Department of Zoology at the University of Oxford, in Oxford, England, in the United Kingdom, and with The Recanati-Kaplan Centre, in Tubney, England, in the United Kingdom. Abigail M. Pointer, Sophia Jingo, Herbert Kasozi, and Tutilo Mudumba are affiliated with the Research on the Ecology of Carnivores and their Prey Laboratory, in the Department of Fisheries and Wildlife at Michigan State University, in East Lansing, Michigan, in the United States. Mordecai Ogada is affiliated with Conservation Solutions Afrika, in Nanyuki, Kenya. Tutilo Mudumba is affiliated with the Department of Zoology, Entomology, and Fisheries Sciences at Makerere University, in Kampala, Uganda.
