# Supplementary material for: Integrating Social Justice into Higher Education Conservation Science
Source: Bioscience. 2022 Mar 30;72(6):549–59. doi: 10.1093/biosci/biac008 (PMC9169897; doi:10.1093/biosci/biac008)
Supplement: biac008_Supplemental_Files [file biac008_supplemental_files.zip › Supplementary_Authorship_Statement.docx]

**Integrating social justice into higher education conservation science**

**Running head:** Social justice in conservation education

**Authors:**

Robert A. Montgomery^1*^, robert.montgomery@zoo.ox.ac.uk

Abigail M. Pointer^2^, pointera@msu.edu

Sophia Jingo^2^, jsophia6@gmail.com

Herbert Kasozi^2^, kasozihe@msu.edu

Mordecai Ogada^3^, mordecai@ogada.co.ke

Tutilo Mudumba^2,4^, tmudumba@gmail.com

**Affiliations**:

^1^ Wildlife Conservation Research Unit, Department of Zoology, University of Oxford, The Recanati-Kaplan Centre, Tubney House, Abingdon Road, Tubney, Oxon OX13 5QL, U.K.

^2^ Research on the Ecology of Carnivores and their Prey Laboratory, Department of Fisheries and Wildlife, Michigan State University, 480 Wilson Road, 13 Natural Resources Building, East Lansing, MI 48824, USA

^3^ Conservation Solutions Afrika, Nanyuki, Kenya

^4^ Department of Zoology, Entomology, and Fisheries Sciences, Makerere University, Kampala, Uganda

**Authorship Statement:**

-Dr. Montgomery led and conceived every component of this study including the course design, the creation of the survey instrument,

-Ms. Pointer contributed to the creation of the survey instrument, participated in the instruction of the course and the data collection, conducted much of the data analysis and figure preparation, and wrote portions and edited the resultant paper.

-Ms. Jingo participated in the instruction of the course and the data collection and wrote portions and edited the resultant paper.

-Dr. Kasozi participated in the instruction of the course and the data collection and wrote portions and edited the resultant paper.

-Dr. Ogada participated in the instruction of the course and the data collection and wrote portions and edited the resultant paper.

-Dr. Mudumba participated in the instruction of the course and the data collection and wrote portions and edited the resultant paper.
