# Supplementary material for: Integrating Social Justice into Higher Education Conservation Science
Source: Bioscience. 2022 Mar 30;72(6):549–59. doi: 10.1093/biosci/biac008 (PMC9169897; doi:10.1093/biosci/biac008)
Supplement: biac008_Supplemental_Files [file biac008_supplemental_files.zip › Supplementary_Materials.docx]

**Supplementary Materials For**

**Integrating social justice into higher education conservation science**

**Appendix A: Animal Personality Test**

The Smalley Trent Personality Test that we implemented to divide students into four learning teams the produce tangible outcomes at the end of each key-performance period. We also gauged the preference of course teams via a ranking.


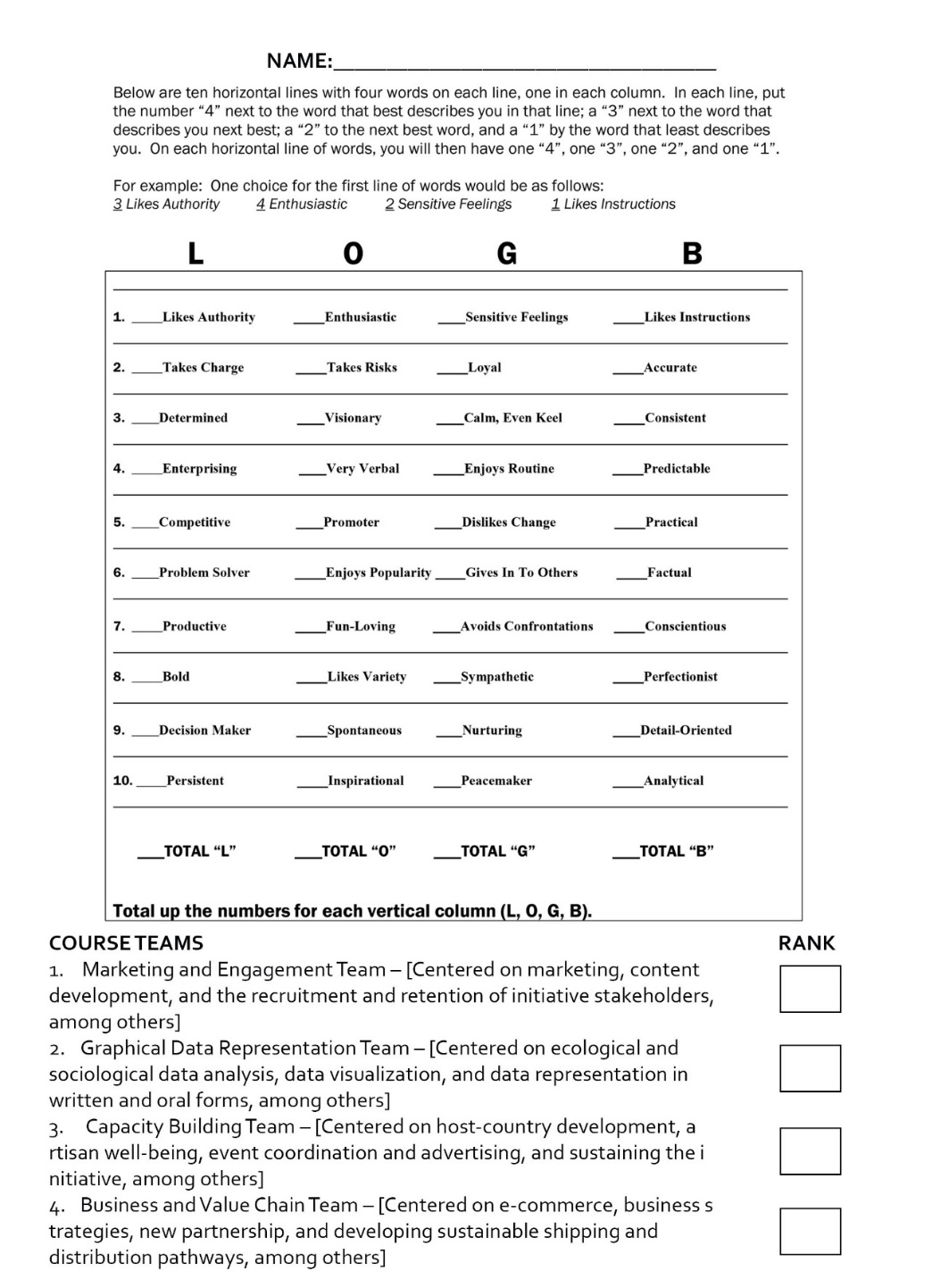


**Appendix B: Survey Instrument**

This instrument depicts both the pre- and post-survey that we implemented among 20 higher education undergraduate students enrolled in our course.

The purpose of this questionnaire is to understand student attitudes and perceptions of wildlife conservation. All responses to the following questions and statements should be viewed in the context of rural East Africa. Herein, we use the term ‘wildlife’ to principally represent large mammals including, but certainly not limited to, species such as lions (*Panthera leo*), elephants (*Loxodonta* africana), and giraffes (*Giraffa camelopardalis*).

The results of this analysis will help Michigan State University to understand the differences in student perceptions and attitudes deriving from experiential learning. It will also help determine whether there are some lessons that can only be learned in novel learning spaces. Thank you very much for filling out this questionnaire!

First, what do you consider to be your level of knowledge on the subject of wildlife conservation in East Africa?

- 1. Very knowledgeable
  2. Somewhat knowledgeable
  3. Slightly knowledgeable
  4. None

The following statements are intended to gauge your values toward ***wildlife***.

1. Please check the statement that most accurately reflects your views.

| Statement | Strongly Agree | Agree | Neutral | Disagree | Strongly Disagree |
| --- | --- | --- | --- | --- | --- |
| Wildlife enhance my enjoyment of natural areas. | [ ] | [ ] | [ ] | [ ] | [ ] |
| We owe it to the global society to preserve wildlife. | [ ] | [ ] | [ ] | [ ] | [ ] |
| We owe it to future generations to preserve wildlife. | [ ] | [ ] | [ ] | [ ] | [ ] |
| Since the public values wildlife, opposition to wildlife conservation deprives the public of their benefits. | [ ] | [ ] | [ ] | [ ] | [ ] |
| When humans and wildlife interact, the outcome is often positive. | [ ] | [ ] | [ ] | [ ] | [ ] |

East Africa features a rich mosaic of human settlements and protected/semi-protected areas (e.g., national parks, wildlife reserves, and conservancies) and is home to a number of species of large mammals (as mentioned above). Given the proximity of humans and wildlife in this region, conflict can occur. Conflict refers to instances in which human-wildlife interaction yields negative outcomes.

Here we are gauging your views of ***responsibility*** when it comes to ***human-wildlife*** conflict. *Costs* are referring to items of personal or economic value to humans.

1. Please check one response to demonstrate the extent to which you agree or disagree with each statement.

| Statement | Strongly Agree | Agree | Neutral | Disagree | Strongly Disagree |
| --- | --- | --- | --- | --- | --- |
| The costs of maintaining wildlife populations in East  Africa should only be paid by the people who wish to conserve them. | [ ] | [ ] | [ ] | [ ] | [ ] |
| Conflict is the result of a few problematic animals (e.g., individual lions, or elephants, or giraffes). | [ ] | [ ] | [ ] | [ ] | [ ] |
| Conflict is the result of a few problematic humans. | [ ] | [ ] | [ ] | [ ] | [ ] |
| When human-wildlife conflict occurs, humans tend to lose. | [ ] | [ ] | [ ] | [ ] | [ ] |
| When human-wildlife conflict occurs, wildlife tend to lose. | [ ] | [ ] | [ ] | [ ] | [ ] |
| Wildlife are a threat to human well-being. | [ ] | [ ] | [ ] | [ ] | [ ] |
| Humans are a threat to wildlife well-being. | [ ] | [ ] | [ ] | [ ] | [ ] |

In the following statements, we are evaluating your views on the appropriate ***response*** to human-wildlife conflict.

1. Please check one response to demonstrate the extent to which you agree or disagree with each statement.

| Statement | Strongly Agree | Agree | Neutral | Disagree | Strongly Disagree |
| --- | --- | --- | --- | --- | --- |
| We ought to tolerate human-wildlife conflict because of the enjoyment the public derives from wildlife. | [ ] | [ ] | [ ] | [ ] | [ ] |
| Human-wildlife conflict is a problem that local land owners should address. | [ ] | [ ] | [ ] | [ ] | [ ] |
| Human-wildlife conflict is a problem that local management authorities should address. | [ ] | [ ] | [ ] | [ ] | [ ] |
| Human-wildlife conflict is a problem that national management authorities should address. | [ ] | [ ] | [ ] | [ ] | [ ] |
| Disturbing (e.g., hazing, relocating) wildlife responsible for human-wildlife conflict is an acceptable reaction. | [ ] | [ ] | [ ] | [ ] | [ ] |
| Injuring and/or maiming wildlife responsible for human-wildlife conflict is an acceptable reaction. | [ ] | [ ] | [ ] | [ ] | [ ] |
| Killing wildlife responsible for human-wildlife conflict is an acceptable reaction | [ ] | [ ] | [ ] | [ ] | [ ] |

In the following statements, we are evaluating your views on the appropriate ***solution*** for human-wildlife conflict.

1. Please check one response to demonstrate the extent to which you agree or disagree with each statement.

| Statement | Strongly Agree | Agree | Neutral | Disagree | Strongly Disagree |
| --- | --- | --- | --- | --- | --- |
| For humans and wildlife to effectively co-exist, humans must adapt. | [ ] | [ ] | [ ] | [ ] | [ ] |
| For humans and wildlife to effectively co-exist, wildlife must adapt. | [ ] | [ ] | [ ] | [ ] | [ ] |
| The knowledge of local people should be valued in wildlife conservation. | [ ] | [ ] | [ ] | [ ] | [ ] |
| Local people are supportive of wildlife conservation. | [ ] | [ ] | [ ] | [ ] | [ ] |
| Local people economically benefit from wildlife conservation. | [ ] | [ ] | [ ] | [ ] | [ ] |
| Local people have the means to prevent conflict with wildlife. | [ ] | [ ] | [ ] | [ ] | [ ] |
| Local management authorities have the means to prevent conflict with wildlife. | [ ] | [ ] | [ ] | [ ] | [ ] |
| Local people should be allowed to manage wildlife in the ways they deem fit. | [ ] | [ ] | [ ] | [ ] | [ ] |
| Local management authorities should be allowed to manage wildlife in the ways they deem fit. | [ ] | [ ] | [ ] | [ ] | [ ] |
| National management authorities should be allowed to manage wildlife in the ways they deem fit. | [ ] | [ ] | [ ] | [ ] | [ ] |
| International management authorities should be allowed to manage wildlife in the ways they deem fit. | [ ] | [ ] | [ ] | [ ] | [ ] |

**Please tell us about your background so that we can better understand your responses. All information is confidential.**

1. Gender:
   1. Male
   2. Female
   3. Non-conforming
2. What is your grade level?
   1. Freshman
   2. Sophomore
   3. Junior
   4. Senior
   5. Other (please specify ______________________)
3. What is your major? ________________________________________________
4. What is your age? ___________

**Appendix C: Animal Personality Test**

Statistically insignificant (at the α ≤ 0.05 level) differences, as measured by paired t-tests, in student responses between the pre- and post-course surveys of the human heritage-centered conservation course.

**
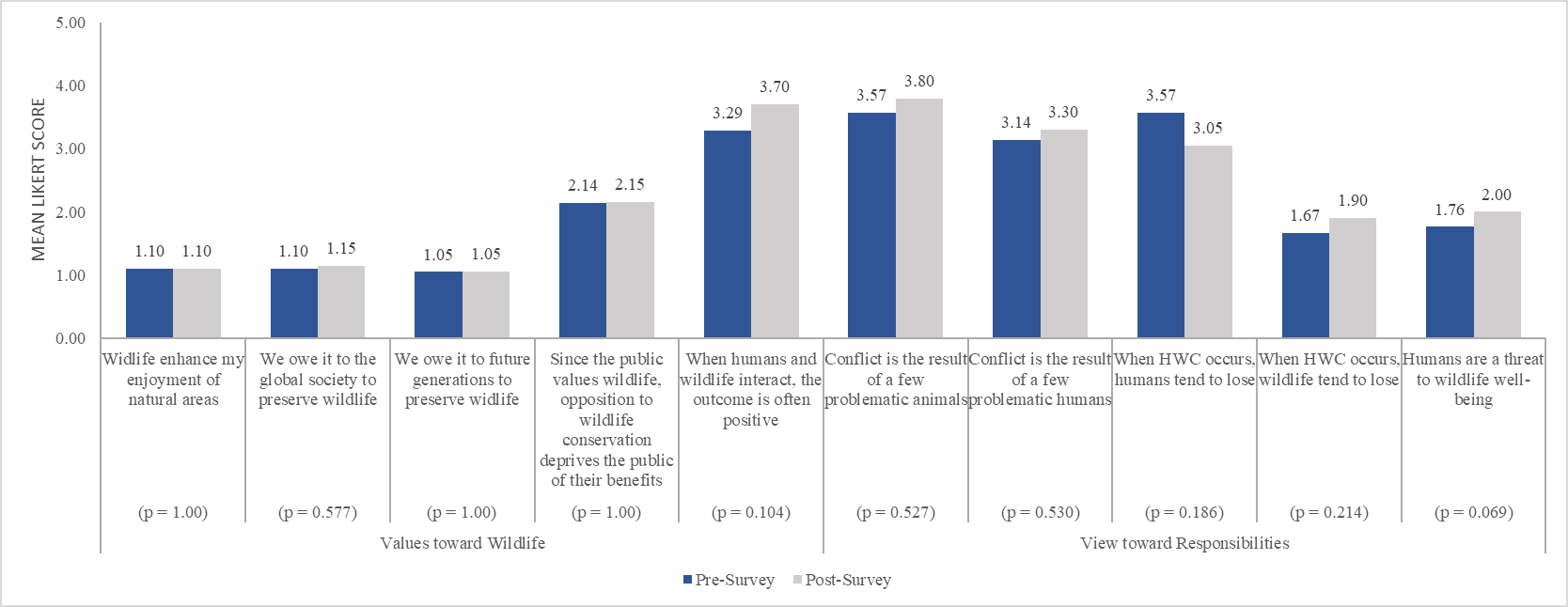
**

**
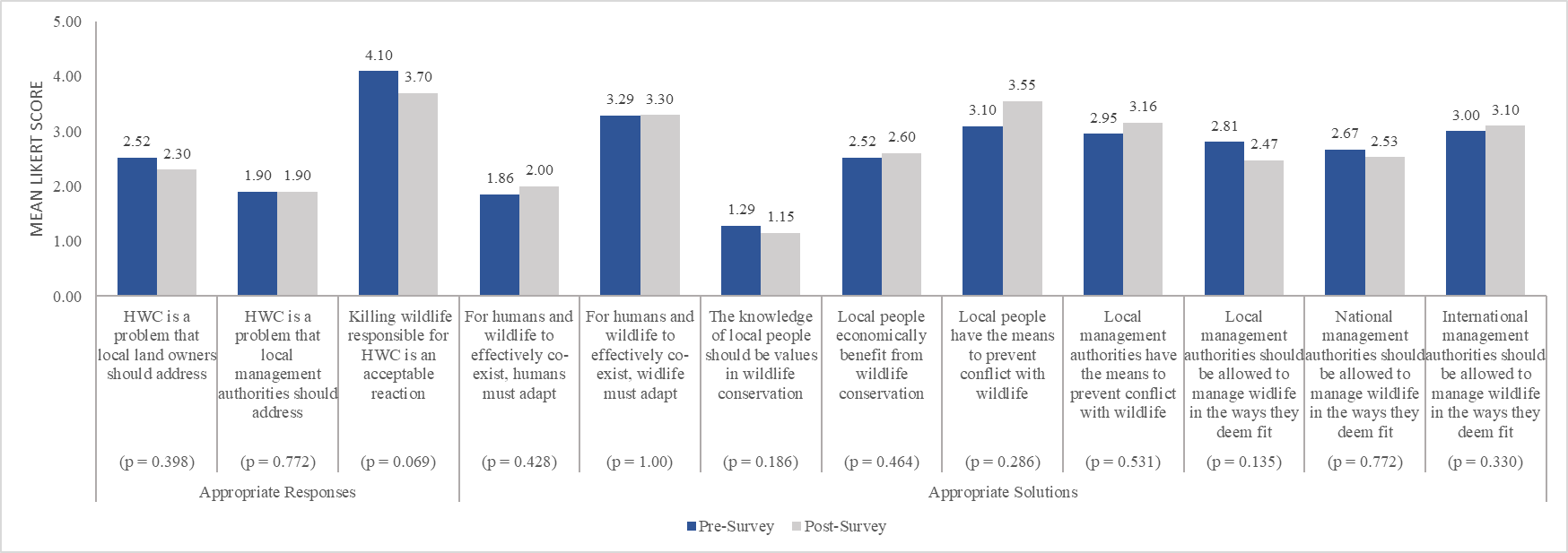
**
